# Facts from Correspondents

**Published:** 1846-01

**Authors:** 


### FACTS FROM CORRESPONDENTS.

We are indebted to *Dr. E. W. Harris*, of Cape Girardeau county, Missouri, for the following interesting communication relative to *Ergot* in cases of flooding and threatened abortion.

"In answer to Dr. Emery's question in the November number of the Western Journal, I will state, that in the year 1826, I was called to see Mrs. J- in the third month of pregnancy, taken six hours before my arrival, with flooding while ironing clothes. The os tincæ was found dilated, membranes protruded, pains regular but weak, and hæmorrhage alarming. Ergot was given with a view of arresting flooding by the expulsion of the uterine contents; in about half an hour the flooding was arrested, the pains ceased, the os uteri closed again, and the woman continued well for two months, when she was, while lifting a heavy burden, suddenly seized with labor pains, and, before my arrival, delivered of twins.

"Since then, I have, on four different occasions, with as many women, witnessed the cessation of *flooding* and *pains*, after the administration of ergot with the view of hastening abortion."

December 1, 1845.

